# Flow-balanced expiration reduces oedema formation in a porcine oleic acid lung injury model

**DOI:** 10.1186/cc9616

**Published:** 2011-03-11

**Authors:** S Schumann, U Goebel, J Haberstroh, M Schneider, HJ Priebe, M Lichtwarck-Aschoff, J Guttmann

**Affiliations:** 1 University Medical Center, Freiburg, Germany; 2 University BioMed Center, Freiburg, Germany; 3 Uppsala University, Uppsala, Sweden

## Introduction

Positive pressure ventilation involves ventilator-controlled inflation of the lungs followed by passive expiration driven by the elastic recoil forces of the respiratory system. In contrast to inspiration where the flow is controlled by the ventilator, expiration is passive, and the only clinically available means of influencing expiration is positive end-expiratory pressure (PEEP). During passive expiration, the flow curve starts with a high peak flow followed by an exponential decay in airflow rate so that typically there is no flow during more than 50% of expiration time. Prolonging the phase of expiratory flow may be expected to be lung protective.

## Methods

Sixteen pigs with oleic acid-induced lung injury were mechanically ventilated for 6 hours with volume-controlled ventilation either without or with flow-balanced expiration. Following insertion of a controllable expiratory resistance into the expiratory outlet of the ventilator, expiratory resistance markedly increased at the beginning of expiration and decreased continuously during the expiration phase.

As a result, the expiratory flow curve changed from an exponentially decaying curve to a balanced flow pattern with lower flow rates at the beginning and higher ones at the end of the expiration phase, thereby achieving complete expiration. Ventilation settings were tidal volume 8 ml/kg, I:E ratio 1:2, RR 15/minute, T_insp _1.5 seconds. Initially PEEP was set at 8 cmH_2_O. During the experiment, PEEP was adjusted to maintain PaO_2 _≥60 mmHg.

## Results

To maintain PaO_2 _≥60 mmHg, after 6 hours of mechanical ventilation PEEP had to be increased from 8 to 13 ± 3 cmH_2_O in the conventionally ventilated animals but to only to 10 ± 1 cmH_2_O in the animals ventilated with flow-balanced expiration (*P *< 0.05). Lung biopsies from animals ventilated without flow-balanced expiration showed more infiltrations and thicker septa compared with those ventilated with flow-balanced expiration (all *P *< 0.05). The wet-to-dry ratio of tissue samples from lungs ventilated with without flow-balanced expiration were higher than those from lungs ventilated with flow-balanced expiration (10 ± 5 vs. 5 ± 4, *P *< 0.05).

## Conclusions

Flow-balanced expiration during mechanical ventilation reduces oedema formation in the injured lung. Reduced expiratory peak flow and increased mean airway pressure during expiration are likely to have contributed to this beneficial effect.

